# Action planning for building public health program sustainability: results from a group-randomized trial

**DOI:** 10.1186/s13012-024-01340-4

**Published:** 2024-02-02

**Authors:** Sarah Moreland-Russell, Todd Combs, Jessica Gannon, Eliot Jost, Louise Farah Saliba, Kimberly Prewitt, Douglas Luke, Ross C. Brownson

**Affiliations:** 1 Prevention Research Center, Brown School at Washington University in St. Louis, 1 Brookings Drive, Campus, Box 1196, St. Louis, MO 63130 USA; 2 Department of Surgery, Division of Public Health Sciences, and Alvin J. Siteman Cancer Center, Washington University School of Medicine, Washington University in St. Louis, St. Louis, MO 63130 USA; 3 Center for Public Health Systems Science, Brown School, Washington University in St. Louis, St. Louis, MO USA

**Keywords:** Sustainability, Action planning, Sustainability plans

## Abstract

**Background:**

Public health programs are charged with implementing evidence-based interventions to support public health improvement; however, to achieve long-term population-based benefits, these interventions must be sustained. Empirical evidence suggests that program sustainability can be improved through training and technical assistance, but few resources are available to support public health programs in building capacity for sustainability.

**Methods:**

This study sought to build capacity for sustainability among state tobacco control programs through a multiyear, group-randomized trial that developed, tested, and evaluated a novel *Program Sustainability Action Planning Model and Training Curricula*. Using Kolb’s experiential learning theory, we developed this action-oriented training model to address the program-related domains proven to impact capacity for sustainability as outlined in the Program Sustainability Framework. We evaluated the intervention using a longitudinal mixed-effects model using Program Sustainability Assessment (PSAT) scores from three time points. The main predictors in our model included group (control vs intervention) and type of dosage (active and passive). Covariates included state-level American Lung Association Score (proxy for tobacco control policy environment) and percent of CDC-recommended funding (proxy for program resources).

**Results:**

Twenty-three of the 24 state tobacco control programs were included in the analyses: 11 received the training intervention and 12 were control. Results of the longitudinal mixed-effects linear regression model, where the annual PSAT score was the outcome, showed that states in the intervention condition reported significantly higher PSAT scores. The effects of CDC-recommended funding and American Lung Association smoke-free scores (proxy for policy environment) were small but statistically significant.

**Conclusion:**

This study found that the *Program Sustainability Action Planning Model and Training Curricula* was effective in building capacity for sustainability. The training was most beneficial for programs that had made less policy progress than others, implying that tailored training may be most appropriate for programs possibly struggling to make progress. Finally, while funding had a small, statistically significant effect on our model, it virtually made no difference for the average program in our study. This suggests that other factors may be more or equally important as the level of funding a program receives.

Trial registration.

ClinicalTrials.gov, NCT03598114. Registered on July 26, 2018.

**Supplementary Information:**

The online version contains supplementary material available at 10.1186/s13012-024-01340-4.

Contributions to the literature

- This study is significant in the development of the first evidence-based training: *Program Sustainability Action Planning Model and Training Curricula*, to increase the sustainability capacity of tobacco control programs.
- The “how” to plan for sustainability or increase programmatic capacity for sustainability has become increasingly more important in the past 5 years as funders have become more concerned with or required sustainability plans, yet few to no resources exist.
- This study highlights future steps in sustainability training research including the need to test other implementation methods (in person vs online) and utility among other public health and chronic disease prevention programs outside of tobacco control.

## Introduction

Public health programs are charged with implementing evidence-based interventions to support public health improvement. For a population to receive the full benefits of implementing an evidence-based intervention, the intervention must be sustained over time. While empirical evidence has established that program sustainability can be improved through training and technical assistance [1, 2], few resources are available to support public health programs in building capacity for sustainability. To date, no evidence-based sustainability training curricula exist to assist public health programs.

Sustainability is the presence of adaptive structures and processes which enable a program to effectively implement and institutionalize evidence-based policies and activities over time [3]. This definition goes beyond the characteristics of a program characteristics and encompasses the organizational and system characteristics of the program. There is a growing body of research on the factors affecting sustainability [1, 4–9], but sparse work has been done to translate the components of program sustainability capacity into practical guides and tools for practitioners to plan for how best to increase their capacity for sustaining evidence-based programs and policies [1, 10].

Only a few conceptual models focus exclusively on the “how” or the programmatic process for building capacity for sustainability. The Dynamic Sustainability Framework offered by Chambers et al. considers the context in which an evidence-based intervention is implemented and operationalized within a system [11]. However, it does not offer an explicit implementation strategy or mechanism based on the alignment of programs with their contexts nor any detailed strategies to actually sustain programs once they have been implemented. May et al.’s normalization process theory explains how new ideas, ways of acting, and ways of working become routinely embedded or normalized in practice settings [12]. It has been utilized in studying program implementation and sustainability [13] and found useful in identifying processes that are likely to enhance sustainability, but again does not offer a mechanism for which programs should engage to improve sustainability.

The Program Sustainability Framework [14], which was utilized for our study, outlines eight domains of sustainability including organizational capacity, funding stability, strategic planning, external environment, partnerships, communication, program adaptation, and program evaluation. These domains have been proven to affect the capacity for sustainability among public health programs [3]; however, understanding how these domains interact to improve program sustainability or how to determine whether success in one domain might improve capacity in other domains is not yet understood. In addition, while these frameworks exist, few are actually referenced in implementation research; few researchers funded by the National Institutes of Health referenced frameworks with sustainability constructs and offered limited information on how they operationalized frameworks (Johnson, 2019).

The “how” to plan for sustainability or increase programmatic capacity for sustainability has become increasingly more important in the past 5 years as funders have become more concerned with or required sustainability plans [15]. For example, the Centers for Disease Control and Prevention (CDC)’s Office on Smoking and Health has required all state-level tobacco control programs which they support (DP15-1509 funding announcement) to design and implement a sustainability plan. However, little has been done to translate the components of program sustainability capacity into practical guides and tools for public health practitioner utilization. Empirical evidence has established that program sustainability can be improved through in-person, hands-on, action-oriented training and technical assistance [1, 2, 16, 17]. Research also highlights the importance of creating an action plan to move sustainability progress forward, and such planning has been shown to predict program survival and post-launch funding [18]; however, to date, no evidence-based sustainability training curriculum exists. Because the state’s tobacco control program (and several other public health programs) funding is consistently at risk of being diminished or eliminated [19, 20], it is important for state programs to engage in some sort of planning for sustainability. In addition, state tobacco control programming involves comprehensive plans, implementation of multiple interventions (health communications, cessation, policy, etc.), and many types of stakeholders including coalitions and state- and local-level interventions. There is therefore an immense need to use the Program Sustainability Framework to understand the various components of these programs and develop an action-oriented planning intervention for improving these program’s capacity for sustainability.

The Plans, Actions, and Capacity to Sustain Tobacco Control (PACT) study sought to build capacity for sustainability among evidence-based state tobacco control programs (TCPs) through a multiyear, group-randomized trial that developed, tested, and evaluated a novel *Program Sustainability Action Planning Model and Training Curricula* [21]. Using Kolb’s experiential learning theory [22], we developed this action-oriented training model to address the internal and external program-related domains proven to impact the capacity for sustainability of public health programs as outlined in the Program Sustainability Framework [3]. This paper aims to evaluate the effectiveness of the *Program Sustainability Action Planning Model and Training Curricula*. To accomplish this, we employed the following research questions:

1. Will the intervention group state TCPs increase their capacity for sustainability more than the control group state tobacco control programs?
2. Does the amount of dosage (i.e., active engagement time) the PACT study utilized have an effect on the sustainability outcomes measured?
3. Is the *Program Sustainability Action Planning Model and Training Curricula* [21] more effective when provided in states with lower tobacco control policy progress than those with higher policy progres

## Methods

The PACT study utilized a multiphase outcome evaluation incorporating a group-randomized experimental design testing the effectiveness of a novel intervention, the *Program Sustainability Action Planning Model and Training Curricula*, to increase the capacity for sustainability among state-level tobacco control programs. This study was approved by the Institutional Review Board of Washington University in St. Louis (reference number 201801196). This study also received approval under Washington University’s Protocol Review and Monitoring Committee. This study was also registered retrospectively on July 26, 2018, as a clinical trial (ClinicalTrials.gov/NCT03598114).

### Intervention development and implementation

The primary goal of the PACT was to provide in-person, manualized training for sustainability action planning and assessment in public health programs. We used a multiphase approach over 5 years (2018–2023) to develop and implement an assessment of the effectiveness of the Program Sustainability Action Planning Model and Training Curricula. In the first phase of the PACT study, the intervention was developed through a rigorous multidisciplinary literature review process and a series of expert consultations. Using Kolb’s experiential learning theory [22], we developed the intervention to address the internal and external program-related domains proven to impact the capacity for sustainability of public health programs as outlined in the Program Sustainability Framework [3]. We used SCOPUS, ERIC (ProQuest), PubMed, Education Full Text, and PsychINFO databases to conduct formative reviews to inform the development and evaluation of the training intervention. Specifically, we performed literature reviews regarding experiential models of learning (i.e., duration and components) and technical assistance (type and duration) to design the intervention. To design the evaluation of the intervention, we conducted formative reviews to assess previous metrics of experiential learning and technical assistance effectiveness. Kolb’s model uses a four-step learning process: (1) concrete learning, (2) reflective observation, (3) abstract conceptualization, and (4) active experimentation [22]. Therefore, we designed a 2-day in-person action-oriented workshop that included (1) didactic presentation regarding program sustainability and the components of the Program Sustainability Framework (concrete learning), (2) discussion of current state program sustainability and state-specific challenges and facilitators (reflective observation), (3) exercises which helped state participants conceptualize and develop program sustainability objectives, and (4) development of a sustainability action plan to be implemented over 3 years. We also consulted with 2 academic experts in sustainability, 2 state tobacco control program directors, and 3 officials from the CDC Office on Smoking and Health to determine the final *Program Sustainability Action Planning Model and Training Curricula* [23]. In the second phase of this study, a multiyear, group-randomized trial was conducted to assess the effectiveness in improving the capacity for sustainability among state-level tobacco control programs (TCP). Ultimately, 11 intervention and 12 control TCPs participated. The *Program Sustainability Action Planning Model and Training Curricula* was delivered to 11 TCPs. The intervention consisted of a 2-day workshop to design a program sustainability action plan, 2 years of tailored technical assistance for implementing the action plan, and sustainability outcome assessment. Participants of the workshops actively engaged in developing state TCP-specific sustainability action plans. Each state action plan outlined 1 or 2 domain-focused objectives, matched with time-specific activities to be shared across stakeholders present. One person at each workshop claimed responsibility for overseeing the implementation process. Sustainability plans were designed to be implemented over the course of 2 years. All Program Sustainability Action Planning Training workshops followed the same structure but were tailored to each state depending on the Program Sustainability Framework domain chosen for the action plan. The 2-day workshop involved the TCP staff as well as a number of stakeholders (i.e., advocates, coalition members, voluntary organizations, grantees, local-level health department staff) actively participating to design a sustainability action plan and develop an implementation strategy. Inclusion of and participation by all stakeholders engaged were an important component of the sustainability action plan development process and ensuring all components of the state TCPs were considered through tailored workshops at baseline and ongoing, robust TA through their 3-year participation [23].

Our main hypotheses for the trial included the following:

1. H1: Intervention group states will increase their capacity for sustainability more than the control group.
2. H2: There will be a positive interaction effect between the group and the amount of dosage, meaning those in the intervention group will benefit more as the dosage increases.
3. H3: The intervention will be more effective for states with lower policy progress (as proxied by the ALA smoke-free score) than those with higher policy progress.

### Participating states and randomization

**Fig. 1:**
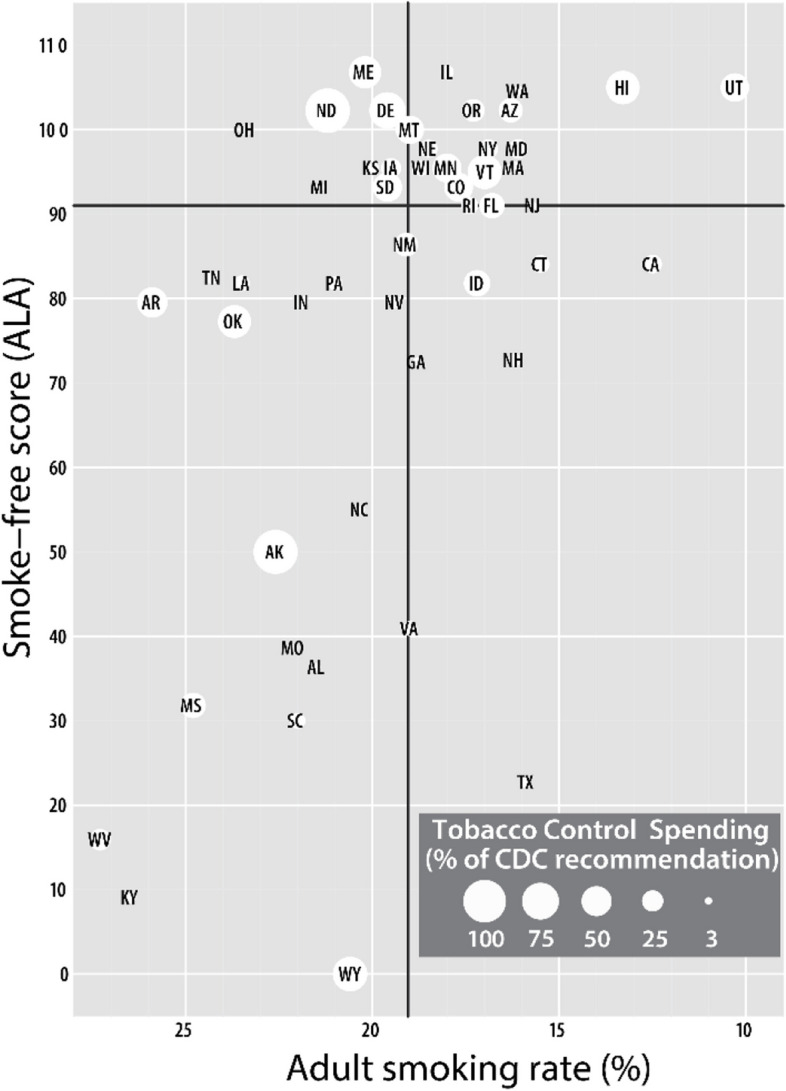
Quadrant stratification for state selection

Our original sample consisted of the 50 US state tobacco control programs. A priori power analyses (at *α* = 0.05) revealed that between 9 (power = 0.8) and 12 (power = 0.9) states per group (control and intervention) would be appropriate. To randomize the two groups, we stratified the 50 states into four quadrants based on the states’ needs (as adult smoking rates) and tobacco control policy environments (as American Lung Association (ALA) smoke-free scores, 2015) [24]. The ALA score is a grade assigned to all 50 US states and the federal government that assesses the state of tobacco control on 4 key tobacco control policies: tobacco control and prevention spending, smoke-free air, tobacco taxes, and cessation coverage. In Fig. 1, smoking rates are on the *x*-axis, and ALA scores are on the *y*-axis. We created the quadrants using the mean scores (black horizontal and vertical lines). The state markers are sized by the percentage of CDC-recommended funding the states spend. We chose 3 states with different degrees of meeting the percentage of CDC-recommended funding [25] from each quadrant. We then chose the closest match (pair) for each state chosen based on the three characteristics displayed. Finally, we randomized states by pairs into the control or intervention group, to balance the 2 groups.

### Measures

Data metrics were defined following recommendations from the advisory board and tobacco control experts and included organizational indicators, Program Sustainability Assessment Test (PSAT) scores, and intervention dosage. Organizational data was collected via record abstraction from annual state-level reports to the CDC Office of Smoking and Health. These reports address fulfillment criteria for the DP15-1509 funding announcement and describe the infrastructure, personnel, and activities of state tobacco control programs in detail. The funding announcements are a requirement of state programs, set by the CDC, to complete yearly reports of progress, goals, and challenges in order to receive federal funding. In addition to the CDC reports, other data was collected via secondary data sources, including the ALA’s annual State of Tobacco Control report [24] and the annual Healthy Americans report issued by Trust for America’s Health. The specific organizational metrics collected are described in a previously published manuscript [21].

In addition, we collected two primary sources of data. First, because it was not feasible to collect all data points through CDC program records, the study team developed a key informant interview tool to collect remaining programmatic information (e.g., staffing capacity and turnover, funding, and achievement of tobacco control goals). The interviews were conducted by phone interview with state program managers or other qualified surrogates (*n* = 21) and lasted 15–20 min. Responses were recorded, transcribed, and reviewed for completeness and accuracy. Any data remaining was collected via an online Qualtrics survey for the convenience of state program managers, who could not complete the phone interview. Only two state program managers or other qualified surrogates completed the online Qualitrics survey—all others (*n* = 21) completed the phone interview.

Data from the Program Sustainability Assessment Tool (PSAT) was also collected at 3 time points (baseline, 1 year post-intervention, and 2 years post-intervention). The PSAT consists of 40 7-point Likert scale items organized into the 8 domains of the program sustainability framework (environmental support, funding stability, partnerships, organizational capacity, program evaluation, program adaptation, communications, and strategic planning). The PSAT was emailed to all stakeholders who participated in the sustainability action planning process in each state. The range of participants per state was 7–15. To complete the PSAT, respondents rated the extent (1, little or no extent; 7, a very great extent) to which the program has or does what the item describes (e.g., “diverse community organizations are invested in the success of the program”). We calculated state-specific means for each of the 40 items. State-specific domain scores were obtained by averaging item scores within a domain. The overall domain scores were obtained by averaging the scores from all participating stakeholders for each domain, and standard deviations were calculated to show variability by state. These scores were used as the outcome in our analyses. The PSAT is a reliable instrument developed to evaluate the capacity for sustainability of public health, social service, and clinical care programs [2, 26].

Active dosage was measured in hours spent in sustainability training, technical assistance, or workshops delivered in-person or virtually. All programs (including control and intervention state TCPs) were given access to online sustainability resources (https://sustaintool.org/psat/resources/ and https://prcstl.wustl.edu/pact-resources/), referred to as passive dosage. A summary of intervention and control group activities can be seen in Table 1.

**Table 1:** Intervention and control group activities

*Activity*	*Frequency*	*Intervention*	*Control*
Initial PSAT meeting (active dosage)	***1 time (baseline)***	***√***	***√***
Annual PSAT assessment	***2 times (years 1 and 2)***	***√***	***√***
Sustaintool.org resource access (passive dosage)	***Ongoing***	***√***	***√***
Technical assistance (active dosage)	***10–12 times (3–4/year)***	***√***	
Two-day sustainability action planning training (active dosage)	***1 time***	***√***	

### Data and analyses

We tested these hypotheses using longitudinal linear mixed-effects modeling using data from the three time points annually in the intervention. We used random effects for the state and fixed effects for all other variables. The main predictors were group (hypothesis 1) and two types of dosage (active and passive). Active dosage was measured in contact hours spent in sustainability training, technical assistance, or workshops delivered in-person or virtually. Passive dosage was measured as a binary where 0 = no resource use and 1 = any resource use, as reported in annual surveys of programs. Other covariates included the percentage of CDC-recommended funding [27] (as a proxy for the level of program resources), ALA smoke-free score [28] (as a proxy for tobacco control policy progress), and program manager tenure as reported in annual surveys to represent program staff turnover or stability. This variable was measured categorically (vacant, less than 1 year, 1–3 years, 3–5 years, and more than 5 years in our annual surveys). In addition, we included interaction terms between the group and each type of dosage (hypothesis 2) and one for the group and ALA smoke-free score (hypothesis 3). The outcome variable was the annual PSAT score. For the model, we also tested for linearity in the model and normally distributed and independent errors. We used the R statistical environment for all analyses.

## Results

Twenty-three of the 24 state programs were included in the analyses; one state dropped out of the study before data could be collected. Descriptive statistics are shown in Table 2. The average PSAT scores increased from 4.6 (sd 0.4) to 4.8 (sd 0.7) for the intervention group and from 4.4 (sd 0.7) to 4.7 (sd 0.7) for the control group. Active dosage hours varied from 1.1 to 7.6 for the intervention group and 0 to 0.8 for the control group. The average ALA smoke-free scores and their variances were similar across the groups and years, as were percentages of CDC-recommended funding. In years 1 and 2, three and five programs in the intervention group took advantage of Sustaintool.org resources, compared to four and one control programs in the same years, respectively. Across years, most programs had managers with at least 1 year of experience: years 0–21 out of 23 or 91%, years 1–19 (83%), and years 2–21 (91%).

**Table 2:** Descriptive statistics for variables used in the regression model with 11 state programs in the intervention group and 12 in the control group for a total of 69 program-years

	**Year 0**		**Year 1**		**Year 2**	
*Continuous variables*	*Mean*	*SD*	*Mean*	*SD*	*Mean*	*SD*
**PSAT**						
1. Intervention	4.6	0.4	4.5	0.7	4.8	0.7
2. Control	4.4	0.7	4.7	0.9	4.7	0.7
**Dosage: active**						
3. Intervention	7.6	1.2	1.5	1.1	1.5	1.1
4. Control	0.8	0.0	0.0	0.0	0.4	0.7
**ALA score**						
5. Intervention	35.3	10.9	34.7	11.4	34.7	11.4
6. Control	32.1	15.4	33.4	15.0	33.6	15.1
**CDC rec. funding (%)**						
7. Intervention	22.9	18.2	21.7	14.9	26.5	25.6
8. Control	27.6	27.6	24.4	26.1	25.5	27.3
*Categorical variables*	*n*	*%*	*n*	*%*	*n*	*%*
**Dosage: passive: none**						
9. Intervention	11	100	8	73	6	55
10. Control	12	100	8	67	11	92
**Dosage: passive: any**						
11. Intervention	–	–	3	27	5	45
12. Control	–	–	4	33	1	8
**PM tenure: vacant**						
13. Intervention	1	9	–	–	–	–
14. Control	–	–	–	–	–	–
**PM tenure: < 1 year**						
15. Intervention	–	–	4	36	2	18
16. Control	1	8	1	8	2	17
**PM tenure: 1–3 years**						
17. Intervention	5	45	–	–	2	18
18. Control	1	8	2	17	2	17
**PM tenure: 3–5 years**						
19. Intervention	5	45	3	27	2	18
20. Control	4	33	1	8	–	–
**PM tenure: > 5 years**						
21. Intervention	–	–	–	–	5	45
22. Control	6	50	8	67	8	67

*ALA* American Lung Association, *PM* program manager

“–” = 0 programs in category

Table 3 contains the results of the longitudinal mixed-effects linear regression model, where the annual PSAT score was the outcome. States in the intervention condition reported significantly higher PSAT scores—on average 1.35 higher—suggesting greater capacity for sustainability after receiving the PACT training. The effects for CDC-recommended funding and ALA smoke-free scores were small but statistically significant indicating that [1] as a program’s funding rose by 1%, its PSAT score would increase by 0.01 (95% CI 0.01–0.02), and [2] as a program’s ALA score increased by 1 (regardless of group), its PSAT score would increase by 0.04 (95% CI 0.02–0.05), all else equal. For context, the average PSAT score increased from 0.1 to 0.3 annually in the study (Table 2). Finally, in states with a higher ALA score, and therefore stronger policy environment, the impact of the intervention mattered less. This effect is explored more below. The remaining variables were not statistically significant at the 5% level. It is also useful to note that we experimented collapsing the program manager tenure variable into three and four categories, and the results were similar and not statistically significant.

**Table 3:** Longitudinal mixed-effects model results

	Outcome: PSAT score		
	Estimate	CI	*p*
Intercept	**2.58**	**1.36–3.80**	** < 0.001**
Group: control		*Baseline*	
Group: intervention	**1.35**	**0.29–2.40**	**0.01**
Year: 0	*Baseline*		
Year: 1	0.31	− 0.08–0.70	0.12
Year: 2	0.34	− 0.00–0.68	0.05
Passive dosage: none	*Baseline*		
Passive dosage: any	− 0.31	− 0.82–0.20	0.23
Active dosage	− 0.06	− 0.44–0.31	0.73
CDC rec. funding (%)	**0.01**	**0.01–0.02**	**0.001**
ALA score	**0.04**	**0.02–0.05**	** < 0.001**
Program manager: vacant	*Baseline*		
Program manager: < 1 year	− 0.01	− 1.03–1.01	0.99
Program manager: 1–3 years	0.21	− 0.75–1.17	0.66
Program manager: 3–5 years	0.43	− 0.63–1.49	0.43
Program manager: > 5 years	0.36	− 0.63–1.34	0.47
Group × passive dosage	0.42	− 0.24–1.07	0.21
Group × active dosage	0.12	− 0.23–0.47	0.50
Group × ALA score	** − 0.05**	** − 0.07 to** − **0.02**	**0.00**
**Random effects and model statistics**			
*σ*^2^		0.15	
ICC		0.4	
N		23	
Observations		69	
Marginal *R*^2^/conditional *R*^2^		0.49/0.70	

Bolded estimates have confidence intervals that do not cross 0 at *p* < 0.05

**Fig. 2:**
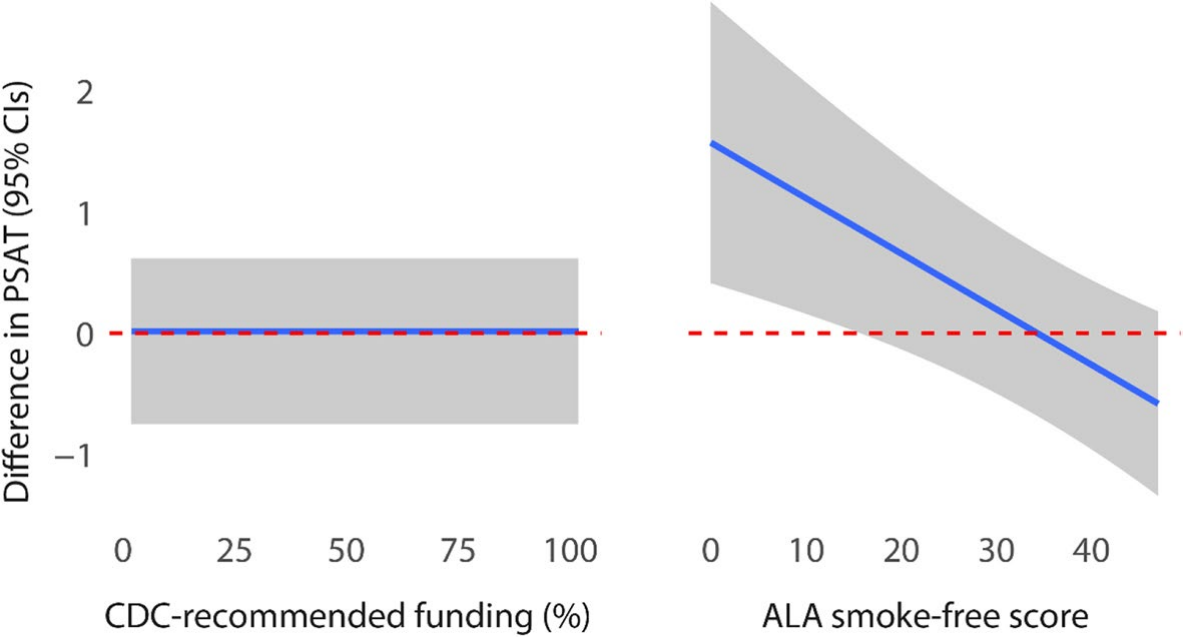
Predicted differences between PSAT scores (intervention minus control) across the ranges of CDC-recommended funding percentage and ALA smoke-free score. All other effects held constant at mean or mode

To complement the results in Table 3, Fig. 2 illustrates the influence of the percentage of CDC-recommended funding that a program receives and the ALA score on a program’s capacity for sustainability when holding all other covariates at their means or modes. The left panel looks at funding and illustrates that the average state program had no influence on the capacity for sustainability from funding levels. The right panel looks at ALA scores, as a measure of strength of tobacco control policy, and indicates that the difference between the groups—or the effect of being in the intervention group—was larger for those programs with relatively low ALA scores. After the program’s scores pass a threshold of around 20, the impact of the score on its capacity for sustainability diminishes.

## Discussion

This study is significant in the development of the first evidence-based training: *Program Sustainability Action Planning Model and Training Curricula* to increase the sustainability capacity of tobacco control programs. There is a growing body of research on aspects affecting sustainability [4, 6–9, 29]; however, little has been done to translate the components of program sustainability capacity into practical guides and tools for practitioner utilization. We developed the *Program Sustainability Action Planning Model and Training Curricula*, based on expert consultation, extensive literature reviews, Kolb’s experiential learning model, and the Program Sustainability Framework. The main goal was to show that tailored training involving experiential learning and action planning could be effective in increasing the capacity for sustainability for recipient programs. We also hypothesized that the amount of dosage defined as mainly active hours of in-person or virtual engagement with the training would positively correlate with increases in sustainability capacity. Finally, we investigated whether the training would be more beneficial to those programs that had made relatively less tobacco control policy progress than others.

We found that the in-person, action-oriented *Program Sustainability Action Planning Model and Training Curricula* was effective for those in the intervention group, regardless of dosage. This suggests that no matter the intensity or frequency of engagement with the training, receiving any amount can influence a program’s capacity for sustainability. Empirical evidence has established that program sustainability can be improved through in-person, hands-on, action-oriented training and technical assistance [1, 2, 16, 17]. Research also highlights the importance of creating an action plan to move sustainability progress forward [18]. Our results further indicate the importance of action-oriented training and technical assistance.

We also found that the training was most beneficial for those state programs that had made less policy progress than others, implying that tailored training may be most appropriate for programs that may be struggling to make progress. States with relatively higher success in policy progress benefited less as demonstrated by the declining difference in sustainability capacity between these programs in our study. However, research consistently indicates that even effectively implemented interventions risk failure when funding, planning, or training ends [6, 29–31]. Given that our study included only 3 years of sustainability tracking, continued research is needed to determine if the difference in policy progress truly serves as a protection factor.

Despite many years of research related to other factors that relate to program sustainability [15], many observers still equate sustainability with funding. We found that while funding had a small, statistically significant effect on our model, it virtually made no difference for the average program in our study. This is not to say that programs do not need funding to survive and sustain themselves, only that other factors may be more or equally important as the level of funding a program receives. For example, the Program Sustainability Framework highlights seven other components important to building capacity for sustainability. Studies have shown that several of these non-funding components from the Program Sustainability Framework, including partnerships, external support, and strong organizational capacity among local health departments [32] and program adaptation, environmental support, and organizational capacity, among state-level chronic disease programs [33] were more important to maintaining program sustainability.

Many also perceive that staff turnover is a major threat to program sustainability. In a scoping review by Pascoe et al. [34], assessing the effects of workforce turnover on program sustainability, 29 of 30 articles related that workforce turnover potentially threatened program components of sustainability, including loss of organizational knowledge, lack of evidence-based program fidelity, and financial stress. In addition, according to the Public Health Workforce Interests and Needs Survey Report (2022) [35], adequate staff capacity is fundamental to providing sustained services in every community. We proxied staff turnover with program manager tenure and found that it had no effect on a program’s sustainability capacity. Again, this is not to claim that high levels of staff turnover might not affect sustainability and believe there is a need to further study the relationship of staff turnover and program sustainability.

### Limitations

Our study has a handful of limitations that deserve mention. There was a state program in the intervention group that dropped out, leaving us with 23 rather than 24 programs. However, power analyses before the study estimated this sample size (at least 11 per group) at between 0.85 and 0.90. We also proxied staff turnover with program manager tenure, due to data availability issues, and these two phenomena may be less related than we assume. Future studies should focus directly on the relationship between sustainability and staff turnover to further illuminate the mechanisms at work.

Finally, while this study analyzed sustainability data over 3 years, we believe to determine the true effectiveness of the *Program Sustainability Action Planning Model and Training Curricula*, programs that utilize the training should track sustainability measures over a greater amount of time.

#### Conclusions and next steps

Research consistently indicates that even effectively implemented interventions risk failure when funding, planning, or training ends [6, 29–31]. In fact, it is estimated that up to 40% of programs end within 2 years of losing funding [36]. Failure to sustain an implemented program negatively impacts communities through loss of trust in public health initiatives and waste of valuable resources [37]. The findings from this study have the potential to improve public health programs by introducing the *Program Sustainability Action Planning Model and Training Curricula* to improve sustainability over time. The benefits of program sustainability will not only benefit the state programs themselves, but also the health of state populations through the continuation of evidence-based public health initiatives. This study’s findings will contribute to the field of implementation science by providing knowledge on “how” to mature and sustain activities over time, thereby achieving the full benefit of significant public health investments. Future research is needed to further validate the results of this study. First, research testing the implementation of the *Program Sustainability Action Planning Model and Training Curricula* within other public health, chronic disease, and healthcare program areas would extend the utility of our work. Testing and development of sustainability training are particularly important within the clinical or healthcare setting. The Clinical Program Sustainability Framework and Tool [38] has been developed, yet training for healthcare sectors to effectively implement the framework and develop sustainability plans is needed. Components of the *Program Sustainability Action Planning Model and Training Curricula* could be revised according to the healthcare setting and tested. Second, given the growth in online-based training because of the COVID pandemic and the need for social distancing, testing the implementation of the *Program Sustainability Action Planning Model and Training Curricula* intervention in an online format could possibly allow for more programs to access the training and for more stakeholders to participate in the planning, especially in more rural states.

### Implications for public health practice

- Implementation of newly funded programs does not guarantee long-term sustainment, so programs and evaluators should take a more exhaustive focus on the factors that influence sustainability
- Targeting tailored trainings at relatively lower-performing programs may conserve resources for programs, evaluators, and implementation scientists.
- Training curricula materials provided to broader public health audiences are associated with increased sustainability of evidence-based practices

### Supplementary Information

**Additional file 1.** Reporting checklist for randomised trial.

## Data Availability

The datasets generated and/or analyzed during the current study are not publicly available due to privacy protections but are available from the corresponding author upon reasonable request.
