# Supplementary material for: Action planning for building public health program sustainability: results from a group-randomized trial
Source: Implement Sci. 2024 Feb 2;19:9. doi: 10.1186/s13012-024-01340-4 (PMC10835962; doi:10.1186/s13012-024-01340-4)
Supplement: Supplementary file 1 — Additional file 1. Reporting checklist for randomised trial. [file 13012_2024_1340_MOESM1_ESM.pdf]

# Reporting checklist for randomised trial.

In your methods section, say that you used the CONSORTreporting guidelines, and cite them as:

Schulz KF, Altman DG, Moher D, for the CONSORT Group. CONSORT 2010 Statement: updated guidelines for reporting parallel group randomised trials

| | | Reporting Item | Page Number |
|---|---|---|---|
| **Title and Abstract** | | | |
| Title | #1a | Identification as a randomized trial in the title. | Title page |
| Abstract | #1b | Structured summary of trial design, methods, results, and conclusions | First page |
| **Introduction** | | | |
| Background and objectives | #2a | Scientific background and explanation of rationale | 1-3 |
| Background and objectives | #2b | Specific objectives or hypothesis | 3, 5 |
| **Methods** | | | |
| Trial design | #3a | Description of trial design (such as parallel, factorial) including allocation ratio. | 3-5 |
| Trial design | #3b | Important changes to methods after trial commencement (such as eligibility criteria), with reasons | 3-5 |
| Participants | #4a | Eligibility criteria for participants | 4 |
| Participants | #4b | Settings and locations where the data were collected | 4-5 |
| Interventions | #5 | The experimental and control interventions for each group with sufficient details to allow replication, including how and when they were actually administered | 3, 4 |

| | | | |
|---|---|---|---|
| Outcomes | #6a | Completely defined prespecified primary and secondary outcome measures, including how and when they were assessed | 5,6 |
| Outcomes | #6b | Any changes to trial outcomes after the trial commenced, with reasons | NA |
| Sample size | #7a | How sample size was determined. | 4,5 |
| Sample size | #7b | When applicable, explanation of any interim analyses and stopping guidelines | NA |
| Randomization - Sequence generation | #8a | Method used to generate the random allocation sequence. | |
| Randomization - Sequence generation | #8b | Type of randomization; details of any restriction (such as blocking and block size) | |
| Randomization - Allocation concealment mechanism | #9 | Mechanism used to implement the random allocation sequence (such as sequentially numbered containers), describing any steps taken to conceal the sequence until interventions were assigned | 5 |
| Randomization - Implementation | #10 | Who generated the allocation sequence, who enrolled participants, and who assigned participants to interventions | 5 |
| Blinding | #11a | If done, who was blinded after assignment to interventions (for example, participants, care providers, those assessing outcomes) and how. | NA |
| Blinding | #11b | If relevant, description of the similarity of interventions | NA |
| Statistical methods | #12a | Statistical methods used to compare groups for primary and secondary outcomes | 6 |
| Statistical methods | #12b | Methods for additional analyses, such as subgroup analyses and adjusted analyses | 6 |

**Results**

| | | | |
|---|---|---|---|
| Participant flow diagram (strongly recommended) | #13a | For each group, the numbers of participants who were randomly assigned, received intended treatment, and were analysed for the primary outcome | 7 |
| Participant flow | #13b | For each group, losses and exclusions after randomization, together with reason | 7 |
| Recruitment | #14a | Dates defining the periods of recruitment and follow-up | 7 |
| Recruitment | #14b | Why the trial ended or was stopped | NA |
| Baseline data | #15 | A table showing baseline demographic and clinical characteristics for each group | State level not individual |
| Numbers analysed | #16 | For each group, number of participants (denominator) included in each analysis and whether the analysis was by original assigned groups | 7 |
| Outcomes and estimation | #17a | For each primary and secondary outcome, results for each group, and the estimated effect size and its precision (such as 95% confidence interval) | 7 |
| Outcomes and estimation | #17b | For binary outcomes, presentation of both absolute and relative effect sizes is recommended | 7 |
| Ancillary analyses | #18 | Results of any other analyses performed, including subgroup analyses and adjusted analyses, distinguishing pre-specified from exploratory | 7 |
| Harms | #19 | All important harms or unintended effects in each group (For specific guidance see CONSORT for harms) | NA |
| **Discussion** | | | |
| Limitations | #20 | Trial limitations, addressing sources of potential bias, imprecision, and, if relevant, multiplicity of analyses | 9 |
| Generalisability | #21 | Generalisability (external validity, applicability) of the trial findings | 9 |

| | | | |
|---|---|---|---|
| Interpretation | #22 | Interpretation consistent with results, balancing benefits and harms, and considering other relevant evidence | 8,9 |
| Registration | #23 | Registration number and name of trial registry | Abstract page |
| **Other information** | | | |
| Interpretation | #22 | Interpretation consistent with results, balancing benefits and harms, and considering other relevant evidence | |
| Registration | #23 | Registration number and name of trial registry | |
| Protocol | #24 | Where the full trial protocol can be accessed, if available | cited |
| Funding | #25 | Sources of funding and other support (such as supply of drugs), role of funders | 10 |

None The CONSORT checklist is distributed under the terms of the Creative Commons Attribution License CC-BY. This checklist can be completed online using https://www.goodreports.org/, a tool made by the EQUATOR Network in collaboration with Penelope.ai
